# Report of the Epidemic Cholera, as It Appeared in the Territories Subject to the Presidency of Fort St. George

**Published:** 1825-07-01

**Authors:** 


*Report of the Epidemic Cholera, as it has appeared in the Territories subject to the Presidency of Fort St. George, drawn up by Order of the Government, under the Superintendence of the Medical Board.* By WILLIAM SCOT, Surgeon, and Secretary to the Board. Quarto, pp. 350. Madras, 1824, with a Map of the Peninsula of India.

———— Wasteful, forth,
Walks the dire Power of pestilent disease.
A thousand hideous fiends her course attend,
Sick Nature blasting, and to heartless woe
And feeble desolation casting down,
The towering hopes and all the pride of man!

THE first reports of this unparalleled scourge, even at the distance of half our planet from us, caused a considerable sensation in this country. There was then no idea of its ever approaching Europe. It has since pushed its advanced guards to the shores of the Black Sea—and it is far from impossible that it may yet spread its destructive ravages from the banks of the Ganges to the banks of the Thames. The late endemic of the Penitentiary, indeed, bore no very faint resemblance to some of the forms of the Indian Cholera, and may possibly have been a harbinger of that dreadful disease.

The report before us is on a similar plan, and constructed of the same kind of materials as the reports from the Presidencies of Bengal and Bombay. "A mass of information has thus been furnished which leaves to the writer of this report little else than the labour of selection and arrangement." As the present report comes down some years later than the reports from the other presidencies, and as the subject of Indian cholera has not been taken up in this series of our Journal, we shall endeavour to convey to our readers a pretty full account of this voluminous document, which can only be known in this country through the medium of periodical publications.

The history of the Indian cholera is not very extensive. An attempt is made indeed to trace accounts of the disease to the mythological writers of Hindoo antiquity, but with very little success. We shall only quote one of these antique gems of Indian medicine. "The *vishuchi* is most rapid in its effects; its symptoms are, dimness of sight in both eyes—perspiration

—sudden swooning, loss of understanding—derangement of the external and internal senses—pains in the knees and calves of the legs—griping pains in the belly—extreme thirst,—*lowness of the bilious and windy pulses*—coldness in the hands, feet, and whole body." Here then we see that *vomiting and purging* (the two most constant symptoms of Indian—or any other cholera) are not mentioned—and consequently there is but very little probability that the description at all applies to the disease under consideration.

It appears very clear, however, that in the time of Bontius, [1629] the cholera was well known in India. His description is incorrect, as to the *incessant and copious discharge of bile*—and so is his etiology of the disease.

"Besides the diseases above treated of as endemic in this country, the cholera morbus is extremely frequent; in the cholera, hot, bilious matter, irritating the stomach and intestines, is incessantly, and copiously discharged by the mouth and anus. It is a disorder of the most acute kind, and, therefore requires immediate application. The principal cause of it, next to a hot and moist disposition of the air, is an intemperate indulgence of eating fruits; which, as they are generally green, and obnoxious to putrefaction, irritate and oppress the stomach by their superfluous humidity, and produce an æruginous bile. The cholera might, with some degree of reason be reckoned a salutary excretion; since such humours are discharged in it, as, if retained, would prove prejudicial. However as by such excessive purgations the *animal spirits are exhausted, and the heart, the fountain of heat and life is overwhelmed with putrid effluvia, those who are seized with this disorder generally die, and that so quickly, as in the space of four and twenty hours, at most.*" iv.

Bontius no where gives a clear account of these *bilious* evacuations. He speaks, indeed, of æruginous bile, but like many others of more modern date, he described what existed in his imagination, rather than what passed before his own eyes.

Next comes Dr. Paisley, in 1774, who dwells on the putridity and acrimony of the bile, without favouring us with any account of the exact colour, taste, or other quality of the evacuations themselves. He speaks of the cholera, however, as being often *epidemic*. Sonnerat, whose travels embrace the period between 1774 and 1781, speaks of a disease on the Coromandel Coast, resembling, in all respects, the late cholera. He mentions it as an *epidemic which reigns*, and states that in one visitation of this disease, "above sixty thousand people, from Cherigam to Pondichery, perished."

In 1781, there appears to have been a pretty extensive spread of the epidemic cholera, as noticed by Jameson in the Bengal report. In 1782, Mr. Curtis gives the best description of the disease that had ever been published, and his writings have been made fully known to the profession through the medium of the remarks made on them many years ago, by Dr. James Johnson, in his work on Tropical Climates. Curtis and Girdlestone have, with their predecessors, fallen into the same error respecting bile. They describe it as flowing in great abundance, and attribute it to the spasms and other phenomena —and yet it was all in imagination? In rummaging over the manuscript reports of this period, Mr. Scot has found descriptions of the disease by Dr. Duffin, Mr. Davis and others—all harping on the old erroneous bilious theory, but all proving the existence of the malady. In 1813, Dr. Johnson published his work, and first denied the etiology and pathology of the disease as maintained by all the writers above alluded to. The correctness of his statements has been amply confirmed by the late epidemics, as well as the justice of his strictures on preceding authors—though these strictures were considered, at that time, as both visionary and heterodox. From that period till the breaking out of the late epidemic, only incidental notices of the disease have been taken.

Our present author, after objecting to any definition which may tend to keep up the bilious etiology of the disease, proposes the adjunct asphyxia, instead of epidemic, biliosa, &c. The *sinking* or arrest of the pulse is a more constant feature in the higher grades of cholera than any other, and on this account he wishes to designate it as *cholera asphyxia.* The charge of pleonasm lies as much against this as against cholera morbus—asphyxia and morbus being both substances and both diseases. And again, asphyxia clearly denotes *privation* not *sinking* of the pulse.* We shall not quarrel with Mr. Scot, however, about the name of the disease, though the *name* has often (as indeed in cholera itself) led to erroneous ideas both in etiology and pathology. To the common or mild forms of the disease, our author, in the following work, applies the term cholera biliosa—to which we have no objection, while *biliosa* is made to appertain to the description *solely,* and is not to include any etiological idea.

"*Symptomatology.* This most formidable disease does not appear to be attended by any premonitory symptoms which can be regarded as being at all peculiar to it; on the contrary, we may safely assert, that it is of sudden invasion: for, though a slight nausea, a laxity of the bowels,

---

* If the term be adopted, the asphyxia should be rendered into an adjective. Thus asphyxia cholera, would surely be more proper than cholera asphyxia.

and a general feeling of indisposition are often found to precede cholera, yet these symptoms are evidently common to many acute diseases; and they are especially frequent in this climate without being followed by any graver ailment. When such symptoms are found to precede cholera, they might with more truth be regarded as indicating merely a certain deranged state of the alimentary organs, a condition of the body which certainly predisposes a person to an attack of cholera.

"The invasion of cholera generally takes place in the night, or towards morning. The patient is sick at stomach, he vomits its contents, and his bowels are at the same time evacuated. This evacuation is of a nature quite peculiar to the disease; the entire intestinal tube seems to be at once emptied of its fæcal or solid matters; and an indescribable, but most subduing feeling of exhaustion, sinking, and emptiness is produced. Faintness supervenes, the skin becomes cold, and there is frequently giddiness, and ringing in the ears. The powers of locomotion are generally soon arrested; spasmodic contractions, or twitchings of the muscles of the fingers and toes are felt! and these affections gradually extend along the limbs, to the trunk of the body. They partake both of the clonic and tonic spasm, but the clonic form chiefly prevails. The pulse, from the first, is small, weak, and accelerated; and, after a certain interval, but especially on the accession of spasms, or of severe vomiting, it sinks suddenly, so as to be speedily lost in all the external parts. The skin, which from the commencement of the disease, is below the natural temperature, becomes colder and colder. It is very rarely dry; generally covered with a profuse cold sweat, or with a clammy moisture. In Europeans, it often partially assumes a livid hue; the whole surface appears collapsed, the lips become blue, the nails present a similar tint; and the skin of the feet and hands becomes much corrugated, and exhibits a sodden appearance. In this state, the skin is insensible, even to the action of chemical agents; yet the patient generally complains of oppressive heat on the surface, and wishes to throw off the bed clothes. The eyes sink in their orbits, which are surrounded by a livid circle: the cornea becomes flaccid, the conjunctiva is frequently suffused with blood: the features of the face collapse; and the whole countenance assumes a cadaverous aspect, strikingly characteristic of the disease. There is, almost always, urgent thirst, and desire for cold drinks, although the mouth be not usually parched. The tongue is moist, whitish, and cold. A distressing sense of pain and of burning heat at the epigastrium are common. Little or no urine, bile, or saliva is secreted. The voice becomes feeble, hollow, and unnatural. The respiration is oppressed, generally slow; and the breath is deficient in heat.

"During the progress of these symptoms, the alimentary canal is very variously affected. After the first discharges by vomiting and purging, however severe these symptoms may be, the matter evacuated is always watery, and in a great proportion of cases, it is colourless, inodorous, and often homogeneous. In some, it is turbid, resembling muddy water; in others, it is of a yellowish or greenish hue. A very common appearance is that, which has been emphatically called the 'conjee stools,' an appearance produced by numerous mucous flakes floating in the watery or serous part of the evacuation. The discharges from the stomach, and those from the bowels, do not appear to differ, except in the former being mixed with the *ingesta.* Neither the vomiting nor the purging are symptoms of long continuance. They are either obviated by art, or the body becomes unable to perform these violent actions; and they, together with the spasms, generally disappear a considerable time before death. If blood be drawn, it is always dark, or almost black, very thick, ropy, and generally of slow and difficult effusion. Towards the close of the attack, jactitation comes on, with evident internal anxiety and distress: and death takes place, often in ten, or twelve, generally within eighteen or twenty hours from the commencement of the attack.

"During all this mortal struggle and commotion in the body, the mind remains clear, and its functions undisturbed almost to the last moment of existence. The patient, though sunk and overwhelmed, listless, averse to speak, and impatient of disturbance, still retains the power of thinking, and of expressing his thoughts, as long as his organs are obedient to his will. Such is the most ordinary course of cholera asphyxia, when its tendency to death is not checked by art.

"A favourable issue is denoted by a rising of the pulse, a return of heat to the surface, inclination to natural sleep, and a diminution or cessation of vomiting, purging, and spasms: these indications being succeeded, after an interval, by the reappearance of fæcal matter in the stools, of bile, of urine, and of saliva." xxi.

Cholera, like other diseases, has presented considerable variety of symptoms. It is curious and important, however, to remark, that varieties in this complaint are not so much observable in different individuals during an epidemic season, as "in what may be termed, *local epidemic visitations.*" Thus, when the disease appears epidemically in a town or district—in the lines of a corps—or the camp of a marching regiment, it may, on one occasion, be distinguished throughout by the paucity of vomiting and the prevalence of purging—or, *vice versa,* spasm may be very predominant in one instance of invasion, and, at another time, it may be little observable. A frequent variety, and the worst of all, is the apparently slight commotion in the system, as evinced by vomiting, purging, pain, spasms, &c. while, on the other hand, a mortal coldness, with arrest of the circulation, comes on from the beginning, and the patient dies without a struggle. This has frequently manifested itself as the prevailing type, and was almost invariably fatal. Fortunately, this dreadful type has seldom lasted long, at any one time; but generally changed into a milder form, or disappeared altogether.

The vomiting, though, generally speaking, a prominent symptom, was entirely wanting, not only in many individuals, but in whole visitations. The purging was a more constant symptom than the vomiting. It was rarely wanting. In respect to the nature of the discharge from the bowels, " by far the most common appearance is that of pure serum, so thin and colourless as not to leave a stain on the patient's linen." Again. " The reappearance of fæcal matter, *especially if tinged with bile,* seldom, perhaps never takes place till the disease has been subdued."*

*Spasm.* No symptom, common to the disease, was more frequently wanting than spasm of the voluntary muscles. It was more frequent in Europeans than in the native patients—and in the robust of either than in the weakly. In the low and most dangerous forms of cholera, spasm was seldom seen. The muscles most commonly affected were those of the toes and feet, and the calves of the legs. Next to these, the corresponding muscles of the upper extremities were affected—then those of the thighs and arms—last of all the muscles of the trunk. " It has been also remarked, that spasmodic twitchings of the muscles have taken place *after death, and have continued for a considerable time.*"

*Collapse.* Of all the symptoms of cholera, none were so invariably present—none, our author thinks, so truly essential and diagnostic, " as the immediate sinking of the circulation." The period at which the marked diminution of vascular action took place was somewhat various. The pulse sometimes kept up tolerably well for several hours—though rarely. It more generally became small and accelerated at an early stage—and, on the accession of spasm or vomiting, suddenly ceased to be distinguishable in the extremities. The length of time in which patients sometimes lived in this pulseless state, was

---

* The following passage in Dr. Johnson's work was ridiculed at the time it was published, as quite a perversion of intellect.

" I appeal to every unbiassed mind—nay, to prejudice itself, whether I have not now proved the truth of that heterodox position with which I set out—namely, that '*an increased secretion of bile,*' so far from being the *cause* of cholera morbus, is, upon the whole, *a favourable symptom*; and, in the very-worst cases of the disease, (mort de chien for example,) *it is entirely absent.*"—*Edition,* 1813.

This, as we observed above, was considered as the paradoxical ravings of a theorist. We leave it to the profession how far its truth has been proved by subsequent observation in India. And we are quite certain that accurate observation of the phenomena of cholera *in this country* will prove that the diseases differ only in degree, and not in their essential nature.—*Reviewer.*

quite extraordinary. Dr. Killet relates a case where the pulse disappeared three hours after the commencement of the attack, and yet the patient survived three days in that condition!

"On the cessation of the spasm or vomiting, and sometimes, apparently, from the exhibition of remedies, the pulse will return to the extremities for a short time, and again it will cease. The superficial veins and arteries are not always collapsed, even when the pulse has ceased. If these vessels be opened in this condition, the contained blood flows out; their walls then collapse, and no more blood can be extracted. There is no authenticated fatal instance of cholera on record, where the circulation has not been arrested, in the extremities at least, long before death took place." xxv.

In the whole of the reports there was but one *apparent* exception to this. We say *apparent;* for we doubt that the disease was cholera at all. It was more probably a *coup de soleil.*

*Thirst.* This and a sense of burning in the region of the stomach, were generally connected together, and very prominent and constant symptoms of cholera. Yet there were exceptions, not only in individuals, but even in epidemic irritations. When thirst was present, it seemed to subdue all other feelings, and the ignorant soldier, as well as the medical man, who firmly believes that cold water is almost certain death, alike eagerly seek and swallow it. Two melancholy instances are recited, where medical officers have exerted their last and utmost efforts to reach, unperceived, even the water of the bathing tub—so intolerable are the pangs of this cruel thirst!

*Skin.* The state of the surface is such as might be expected from the state of the intestinal canal and the subdued circulation. It is cold, generally clammy—and often covered with profuse cold sweats. In this respect, however, there were varieties. The skin is sometimes observed to be dry, though cold—sometimes of natural, nay, of preternatural warmth. An increase of temperature was repeatedly observed to take place just before death—confined, however, to the trunk and head. In almost all cases this *partial* development of heat was found to be a fatal symptom. It was entirely unconnected with any restoration of arterial energy, or improvement in the function of respiration. The skin, when much collapsed, becomes insensible, even to the action of chemical agents—hence, the usual vesicatories fail in producing any effect.

"It is certain, that in a body *but just dead,* the application of boiling water will vesicate readily; and, if the accuracy of the observation respecting its non-vesicating power in advanced stages of cholera be established, we must infer, that there is less vitality in the skin in such cases, the patient being still alive, than in that of a body *recently* dead of some other disease." xxvii.

At a very early stage of cholera leeches could procure little or no blood from the skin.

*Countenance.* That remarkable shrinking of the features, which acquired the emphatic term of "true cholera countenance," appeared in every case not quickly cut short by medicine. This expression of countenance, which conveys too truly that of death itself, could not be mistaken—and it was evident that a similar shrinking took place throughout the limbs and all projecting parts of the body. The eyes not only became dim, and vision imperfect, but an actual film was sometimes seen over the cornea. The abdomen was sometimes tumid, but much more frequently drawn towards the spine.

*Sensorial Functions.* These, as has been already observed, were surprizingly free from disorder, as compared with other vital functions. In this respect, cholera bears a considerable affinity to tetanus and even hydrophobia.

*Recovery.* "When medical aid is early administered, and when the constitution is otherwise healthy, the recovery from an attack of cholera is so wonderfully rapid, as perhaps to be decisive of the disease being essentially unconnected with any organic lesion." Among the natives, this rapidity of recovery was more remarkable than among the Europeans, where there was, of course, a greater proneness to inflammatory diathesis. The most frequent sequelæ of cholera, were affections of the intestines, brain, liver, and stomach.

*Urinary Secretion.* This, like all the other natural secretions, appeared to be very generally suspended. When not so, the urine was commonly limpid and clear, though in very small quantity—"a curious phenomenon, considering the probable state of the blood under such circumstances." The reappearance of this secretion, when it had been suspended, was always a most favourable omen.

*State of the Blood.*

"No symptoms of cholera are so uniform in their appearance and progress, as those connected with the blood and its circulation. Although the reports, in general, afforded ample reference to this point, it still appeared to the Medical Board to be one of such importance in the pathology of the disease, that a circular letter was addressed to about thirty Medical Officers, who were supposed, from their experience in the treatment of it, to be best qualified to afford information. Attention was especially directed to the following considerations: first, the influence which the state of the blood, in those affected with cholera, might be supposed to have in producing some of the symptoms: second, the colour of the blood abstracted from a vein in a person affected with cholera: third, the colour of the blood after a certain quantity had been taken, and the effect which any alteration of colour might have on the condition of the patient: fourth, if arteriotomy had been practised, the colour of the arterial blood in cholera: and, lastly, the period, from the first attack of the disease, at which blood was abstracted. It is established by the replies to this letter, as well as by an immense mass of concurrent evidence, that the blood of persons affected with cholera, is of an unnaturally dark colour and thick consistence. These appearances are very uniformly expressed by the terms, dark, black, tarry, in regard to colour; and by thick, ropy, syrupy, semicoagulated, in respect to its consistence. The change in the condition of the blood is likewise fully proved to be in the ratio of the duration of the disease; the blood, at the commencement, seeming to be nearly, or altogether natural, and more or less rapidly assuming a morbid state as the disease advances. Some very rare cases are recorded where, however, this morbid state of the blood was not observable, although the disease had been for some time established: and instances have occurred where the blood flowed readily, sometimes little altered, where, nevertheless, death ultimately ensued. The abstraction of blood has been found by all practitioners to be very difficult and uncertain; and the uncertainty has been variously imputed to the feebleness of the circulation, to the thick consistence of the blood, and to the combined operation of these causes. The blood drawn from patients suffering under cholera, is stated to be generally very destitute of serum, never to exhibit the appearance of buff, and to be generally disposed to coagulate quickly. Several instances, however, have occurred, where the coagulation was slow and imperfect. A great majority of the reports state unequivocally that, after a certain quantity of dark and thick blood has been abstracted from a patient under cholera, it is usual for its colour to become lighter, its consistence to become less thick, and for the circulation to revive: such appearances always affording grounds for a proportionably favourable prognosis. In many instances, however, no such changes have been observed to accompany the operation of bleeding, while yet the result was favourable. The blood is generally found to be less changed in appearance in those cases of cholera which are ushered in with symptoms of excitement, than where the collapsed state of the system has occurred at an early period. The blood has been occasionally found, on dissection, to be of as dark a colour in the *left*, as in the *right* side of the heart; affording reason to believe that in the whole arterial system it was equally changed. The temporal artery having been frequently opened, the blood was found to be dark and thick, like the venous blood: but it would appear that this operation has not been performed in general, until the attempts to procure blood from the brachial or jugular veins had failed: little or no blood could be obtained, the artery merely emptying itself in a languid stream, not in a jet, and then collapsing. An instance is stated where the surgeon, despairing of other means, cut down upon the brachial artery, but so completely had the circulation failed, that no blood flowed. When reaction has been established, the blood occasionally shews the buffy coat." xxx.

*Terminations of Cholera.* It was the declared opinion of many practitioners who had to cope with this disease, "that its tendency to death was so great as never to be counteracted by the unaided efforts of Nature." The same opinion was no less evidently implied by the observations of *all*, "that the delay of but a few hours placed the patient beyond the reach of art—for hours in this disease were as days in any other." There were not wanting, men who, from a train of unfortunate cases, or affectation of singularity, maintained that remedial agents had no power over the disease, and that recoveries were owing to Nature, not to art. This opinion, we think, is negatived by the clearest evidence. Among the natives, where remedial aid was not procured, or ignorantly applied, recovery was rare indeed. The Ameen of Ganjam wrote thus:—"the people who get the cholera morbus never recover—death to them is certain." The resident at Hydrabad stated "that he feared every case treated by the natives proved fatal." The family of a wealthy Nair, in Travancore, consisting of 19 people, were all, save one, cut off in a few hours. Another family of five all died. Mr. Searle, at Manantoddy, stated that, of 28 villagers attacked with the cholera, 26 died. The other two recovered by his assistance. "Death may, therefore, be said to be the ordinary termination of cholera."

*Diagnosis.* This is not very difficult. Our author divides the disease into two species—that which appears *with*, and that which appears *without* bile. We have no hesitation in affirming that these are only two *grades* of the same affection. They appear cotemporaneously in the same epidemic—in the same regiment—nay, in the same family.

*Post-mortem Appearances.* These have been very extensively ascertained in the bodies of Europeans; but in a very limited degree in the bodies of natives, from the strong prejudices against dissection. The post-mortem examinations so generally practised in Anglo-Asiatic hospitals were attended with some disadvantages, owing to the heat of the climate, and the necessity of interring the corpse a few hours after death—thus leaving very little time for minute investigation of diseased structure. These disadvantages, however, are not without some advantages. The circumstances above stated rendered it necessary that necroscopic examinations should be commenced almost immediately after the vital spark became extinct—a matter of no mean importance in pathology. But it ought to be borne in mind that "dissections have been chiefly made on the bodies of European soldiers—a class of men acknowledged to be peculiarly liable, in this climate, to visceral disease of all kinds." Under these circumstances, as Mr. Scot properly observes, dissection reports should be viewed with care, in reference to the general state of morbid bodies—and with the most attentive consideration of the precise import of the terms employed. We shall give a general view of the *post-mortem* researches of our Indian brethren, in the words of Mr. Scot himself.

"The external appearance of European subjects, who have sunk under cholera, closely resembles that which has been noticed as taking place during life. The surface is livid, the solids are shrunk, the skin of the hands and feet is corrugated. There seems no sufficient evidence of any uncommon tendency in the body to putrefaction after death, nor of any characteristic fœtor from the abdominal cavity. No particular morbid appearances have been found in any of the cavities of the body, which are lined with *serous membranes*, or in these membranes themselves. The cavities of the pleura, of the pericardium, and of the peritoneum, have almost uniformly been found in a natural state; or the deviations from that state have manifestly had no connection with cholera. The surfaces which are lined, or covered with *mucous membranes*, have, on the contrary, very generally exhibited signs of disease. These will be noticed, as the organs connected with them come to be mentioned.

"The lungs have not unfrequently been found in a natural state, even in cases where much oppression of respiration had existed previously to death. Much more generally, however, they have been found either to be gorged with dark blood, so that they have lost their characteristic appearance, and have assumed more that of liver or spleen; or they have been found to be in the opposite state; that is, collapsed into an extremely small bulk, and lying in the hollow on each side of the spine, leaving the cavity of the thorax nearly empty. This appearance has been so remarkable as to induce Dr. Pollock, of H. M's. 53d regiment, to conceive, that it could only be produced by the extrication of a gas within the cavity of the pleura, capable of overcoming the atmospheric pressure. It is understood, however, that opportunities were had of piercing the thorax of the dead body under water, and that no gas was extricated. As there appears to have been an absolute vacancy in the cavity of the pleura, that is to say, the lungs did not by any means fill it, it would seem that that viscus had exerted a contractile power, adequate to overcome the pressure of the atmosphere. The blood found in the lungs has been always very black. The heart and its larger vessels have been found to be distended with blood, but not so generally as the apparent feebleness of their propelling power, and the evident retreat of the blood to the centre, would have led us to expect. The right auricle and ventricle being gorged with blood, is nothing peculiar to cholera: but some dissections have shewn the left cavities to be filled even with *dark* or *black* blood, which we may reckon as a morbid appearance more peculiar to it. In the abdominal cavity, the peritoneal coverings of the viscera, being *serous membranes*, present in general but little deviation from the healthy state: occasionally, indeed, the morbid accumulation of blood in the vessels of the viscera, imparting an appearance of turgidity and blueness, is evident on their exterior surfaces. We also find them bearing marks of inflammation, especially where the patient may have lingered long before death. In other cases, the whole tube has had a blanched appearance, both externally and internally. The stomach and intestines generally preserve their ordinary volume. The appearance of the omentum is not sensibly affected in cholera. The stomach is found to be so variously affected as to destroy all grounds for pathological reasoning. It is very rarely found empty or much contracted after death, nor has any appearance of spastic stricture of the pylorus been often detected. It has, however, sometimes occurred. Its contents appear to be chiefly the ingesta in an unaltered state: in some cases, greenish, or yellow, or turbid matters are found. The stomach has been said to have been found 'lined with calomel.' Various appearances, either of active inflammation, or a congested state of the vessels, have been noticed, sometimes in one part, and sometimes in another. The parts seem as if they were sphacelated, thickened, softened, and friable; and, in short, exhibit so great a variety of appearances, from a perfectly natural state to the most morbid, that no particular light is thrown by them on the disease.

"The intestinal tube is sometimes collapsed, but oftener found to be more or less filled with air; distended in some parts into bags or pouches, containing whitish, turbid, dark, or green-coloured fluid: and, in others, presenting the appearance of spastic constriction. The latter, however, is not common. No fœcal or other solid matters are found in the intestines; but, very commonly, large quantities of the conjee-looking fluid, or of turbid serous matter. The duodenum, and, occasionally, the jejunum, have been found loaded with an adherent, whitish, or greenish mucus; at other times they have been found seemingly denuded of their natural mucus: and often perfectly healthy. Traces of bile in the intestines, or of any substance apparently descended from the stomach, are exceedingly rare. Sanguineous congestion, and even active inflammation, are stated to be more common in the bowels than in the stomach; but, on the other hand, instances are very numerous where no such indications have been detected. The thoracic duct is stated to have been empty of chyle. The liver has been commonly found to be gorged with blood, but not always: it is an organ usually very vascular: and it would probably demand a nicer discrimination than has been bestowed on the subject, to distinguish the degree of congestion in which it is naturally left by the settling of the blood after death in ordinary diseases, from that which has been observed after an attack of cholera. The gall bladder has almost universally been found to contain bile, and, in the great majority of cases, even to be completely filled with it. As is usual with this secretion, in cases of retention, it is of a dark colour. Very different states of the gall ducts have been described: cases of constriction and impermeability, seeming to be equally numerous with those of an opposite character.

"The urinary bladder is found, we may say universally, without urine, and very much contracted. The lining or mucous membranes of the bladder and ureters have been found coated with a whitish mucous fluid. The smallness of the bladder after death has been generally adduced in proof of great spasm: but it is not unfrequently found to be equally small after death from other diseases: and it seems the nature of that organ, when it contains no urine, to contract, so as to leave no cavity. Dr. Baillie, in his morbid anatomy, thus notices this fact. 'The bladder is also found contracted to such a degree as hardly to have any cavity. This is generally not to be considered as a disease, but simply as having arisen from a very strong action of the muscular coat of the bladder previously to death.' The appearance of the spleen, which is so various under the ordinary conditions of the body after death, has indicated nothing that can be mentioned as belonging to cholera. The vessels of the mesentery have been very generally found to be uncommonly full of blood.

"In the head, appearances of congestion, and even of extravasation, have been frequently observed; but not so uniformly nor to such extent as to require any particular notice. Only one case has been given where the state of the spinal marrow was examined: and, in that, indications of great inflammation were detected in its sheath: the case, however, was, in some degree, a mixed one.

"From this general view of the appearances found on the dissection of the bodies of persons who have died from cholera, it is manifest that the information thence derivable, is, in a pathological view, of a negative nature only. It is nevertheless of consequence, in a practical sense, especially in treating the sequelæ of cholera." xxxiv.

*Ratio Symptomatum.* It has been seen that the *natural functions* are all, in turn, disordered—but no one *invariably* so. Of the *vital functions*, that of the heart and blood-vessels may, he thinks, be stated to be *invariably affected* in the asphyxic cholera. The function of the lungs must suffer, of course, along with that of the heart. This last inference is proved by the state of the blood, which has been already described—and the reduction of temperature. The animal and the sensorial functions appear to be least of all affected in this disease. It has been shewn by dissection that the *serous* membranes are not necessarily affected in cholera; but that the *mucous* membranes, including the skin, are, in some part or other, invariably affected. There is no proof, Mr. Scot thinks, "that all the glandular secretions, as has been commonly supposed, are *entirely* suspended in cholera." "It is the concurrent opinion, however, of all medical men who have treated cholera, that the natural secretions, if not absolutely suspended, are, at least, very greatly interrupted." While this is the case, there are other discharges established, with the nature of which we are but little acquainted. We have no chemical analysis of the fluid thrown out by the skin, the stomach, or the intestines—and we cannot say whether it be a morbid secretion peculiar to the disease, or merely an increase of the natural secretions. We need not detail Mr. Scot's further observations on the ratio symptomatum. They all go to shew, in our opinion, that a deadly sedative agent presses on the vital organs, especially the nervous and vascular systems, suspending or deranging their functions. The subordinate links in the chain of causation are of minor importance. They must depend on the great primary morbid impression on the systems above-mentioned.

*Predisposing Causes.* All accounts agree that the young, the healthy, and the robust, were the least liable to cholera. People, on the other hand, who had been debilitated by disease, or by the remedies employed for the removal of disease, were unusually susceptible of the epidemic. One attack was far from conferring on the patient an immunity from a second. On the contrary, it seemed rather to give a greater predisposition to it. The *exciting* causes were the same as excite most other diseases, as errors in diet, fatigue, atmospherical vicissitudes, depressing passions, &c.

It is proved by the various reports that the cholera has appeared, and was equally virulent, during all states of the atmosphere—amidst all diversities of the surface of the country—and under every variety of the circumstances of the people. It was found, however, that a corps under march, ran a greater risk of being attacked by cholera, than a corps in quarters. This was evidently not so much owing to the fatigues and privations of the journey, as to the traversing certain inauspicious spots. There is no instance on record, of a ship from Europe having a single case of the disease, until she communicated with the land:—but there are many examples of cholera appearing on board of ships sailing from the continent of India. This in itself, is a proof either that the disease is produced by terrestrial influence or contagion. The latter is very improbable. But more of this anon.

*Influence of the Atmosphere.* It is abundantly proved that cholera has not been confined to any particular tract of country or period of time—although it has been infinitely more prevalent at some periods and places than at others. The disease has been capable of exerting its influence in every state of atmosphere, so far at least as is evident to our senses or determinable by instruments. It has now continued for nearly seven years, during which time it has spread to China in the east, the Mauritius in the south—to Persia and Turkey in the west. There is nothing hitherto ascertained respecting cholera which can enable us to fix the limits of its range.

"We are thus furnished, on one hand, with arguments to prove that the cause of cholera exists in the atmosphere; and, on the other hand, with no less powerful reasons for thinking, that the morbific influence may be something arising from the soil, not generally and equably diffused through the air. Ships arriving in the Indian seas ought to suffer under the epidemic influence of the air, if such influence really existed; but, it is certain, on the contrary, as already stated, that no instance has ever been recorded of the crew of a ship suffering from cholera, until the vessel had come into communication with the land." xxxix.

A change in the electricity of the atmosphere, especially a diminution of it, has been assigned by Mr. Orton as the immediate cause of cholera. According to this gentleman, the electric fluid is the great agent of life—the primum mobile of the brain and nervous system. This idea is already quite obsolete, and ever had but a very limited number of supporters.

"Now, though we have certainly not been able to ascertain by experiments, the precise electrical state of the atmosphere during attacks of cholera, it is sufficiently manifest, that the disease has raged with equal violence under every sensible condition of the weather: and in fact, a great number of the attacks have taken place, when the sky was clear and serene, and when every appearance indicated an undisturbed state of the electrical fluid. But, if a deficiency of electricity be the true and sole proximate cause of cholera, which the theory upholds, it seems objectionable to limit its influence to epidemic attacks: for each individual case, whether sporadic or otherwise, must be equally the effect of this proximate cause. Sporadic cases have, however, been too numerous, and too uniform in their occurrence, for some years past, to warrant the conclusion, that they are connected with any particular state of the electricity of the atmosphere." xli.

Of sol-lunar influence—damaged rice, &c. we need not here speak. The latter is clearly not the cause of cholera—and of sol-lunar influence, as a cause of this dreadful epidemic, there is nothing like proof.

*Contagion.* "If this question," says our author, "could have been decided simply by the opinions of a majority of medical men, it would have been already set at rest *against the doctrine of contagion* or infection"—"for there are few subjects, perhaps, on which so little diversity of sentiment has existed." Still there were a few who advocated the contagious nature of cholera, and our author has deemed it necessary on that account to go into the question rather minutely—more so, indeed, than there was occasion for, in our humble opinion. "It is not contended," says our author, "by those who embrace the doctrine of infection, that cholera has not arisen spontaneously, as well at the present as in former times; nor is it considered that this circumstance affects the question." Their doctrine then is that of contingent contagion—which narrows the question very much. Indeed we see so many diseases, as for instance erysipelas, taking on an occasional character of contagion, that it would probably not be safe to deny, *in toto*, the possibility of contingent contagion in the epidemic under consideration. We cannot here enter into the facts and arguments, pro and con, which Mr. Scot has arrayed in opposition to each other. The question is far from being definitively decided either way—though the preponderance of the anticontagionist arguments is overwhelming.

*Proximate Cause.* We know not the proximate cause, that is, the *intimate nature*, of any disease—or hardly of any disease—and that of cholera morbus is not likely to prove an exception to the general rule. We shall therefore dismiss the subject at once, and proceed to one of more importance.

*Treatment.* In no disease has the sovereign efficacy of numerous specifics been more vaunted—in none have they been more frequently inefficient, than in cholera. While the general tendency of the disease was almost invariably towards a fatal termination, there was yet a certain stage, at which its progress might, in very many instances, be arrested by the most opposite, and sometimes by the most trifling remedies. The earliest movements in cholera, especially as it first manifested itself, were generally confined to irritation of the stomach and bowels —"and in this stage it is unquestionable that the mere exhibition of an anodyne, a cordial, or an antispasmodic medicine was sufficient, in numberless instances, to stop the progress of the disease, and to effect a cure." After the first epidemic sweep, however, of the cholera, it was universally acknowledged by practitioners, that the remedies so successful in their former practice, were almost totally unavailing in the subsequent visitations.

*Opium.* No medicine has been so universally used as this, and none, perhaps, has so generally retained its reputation. It was more successful, however, among the natives than among the Europeans, from their greater susceptibility to the action of medicines.

"While, as an anodyne and antispasmodic, the efficacy of opium in the cure of cholera has been proved by experience to be great, it is painful to be obliged to acknowledge, that in many apparently promising cases, where, even, its narcotic effect was evident, it had no ultimate effect in warding off a fatal termination: and in the advanced stages of the disease, whether it was first exhibited at that period, or whether its use may have been begun earlier, and then continued, its efficacy in restoring the depressed or suspended functions, like that of all our other remedies, is extremely uncertain. Those cases may, with most confidence, be chiefly trusted to the effect of opium, in which the primary symptoms are seated, apparently, in the stomach, as indicated by vomiting, and spasmodic pain in that region; and in the intestines, as indicated by violent purging, and painful contractions of the abdomen. Its effects are more uncertain, where the affection of the stomach is obscure; where there is a moderate, but insidious purging; where there is great sense of heat in the epigastrium, and in every case where collapse has come on." Iv.

Passing over æther, ammonia, spirits, wine, and other diffusible stimuli, we come to

*Calomel.* This was almost as universally given as opium, and as indiscriminately. It was administered for various indications, and with various views, according to the practitioner's opinions respecting the nature of the disease. Mr. Scot does not appear to be favourable, on the whole, to the administration of calomel, at least in the early stage of cholera.

"The success of those practitioners, who did not use calomel in the primary treatment of cholera has been fully as great as that of those, who did: while the arguments, distinctly brought forward by various Medical Officers against its *early exhibition*, appear decisive against the observations which have been offered in support of the practice. Calomel has unquestionably a powerful effect in exciting the biliary system, and in this view, its exhibition is highly necessary: but the suppression of the excretion of bile being only a link in the common chain of symptoms, and its partial, or occasional removal, or even its total absence, having been proved to be of little consequence in the general course of the disease, to attempt to excite it by particular means may be considered as premature and injudicious. Whenever a favourable change takes place, indicated by the renewal of the *ordinary* functions, then the exhibition of the appropriate stimulus seems to be clearly indicated, and not till then." lvii.

*Blood-letting*. This remedy was first proposed by the editor of this Journal, from observing its effects in one or two sporadic cases of cholera in the island of Ceylon, more than twenty years ago. It will be seen, from the following extract, that it has been an important measure of late, in the treatment of this terrific malady.

"The abstraction of blood, unless as an anti-spasmodic, is a remedy so little indicated by the usual symptoms of cholera, that its employment in the cure of this fatal disease has afforded a signal triumph to the medical art. It requires no common effort of reasoning or reflection to arrive at the conclusion, that, when the powers of life appear depressed to the lowest degree, the pulsation of the heart all but extinct, the natural heat of the body gone, and the functions of the system suspended and incapable of being revived by the strongest stimulants, the abstraction of blood might yet prove a remedy against a train of symptoms so desperate. Bleeding was, no doubt, first employed in cases where there was much spasm, and where the powers of the system had not much declined: the relief was generally obvious and immediate, and the practice in such instances was thus established. Dissections having frequently shewn a loaded state of the vessels of the viscera, and apparent inflammation of their mucous membranes, venesection was also adopted to obviate these conditions, and naturally enough: but the employment of bloodletting, without reference to either of these states, and as a remedy for collapse in cholera, must have been the result of reasoning and reflection, founded on the general principles of the science; a result highly honorable to the profession; and, as we shall endeavour to shew, of the utmost practical importance in the cure of the disease.

"We have no precise information regarding the manner, in which venesection was performed in general; although, in such a disease, this seems a matter of very considerable consequence. It is remarkable, that in a disease like cholera, syncope should be so rare a symptom. When it is brought on by venesection, it is generally favourable, which may most probably be imputed to its being employed during violent spasm, and before any sinking has taken place. We can then readily understand, that the free evacuation of blood, which may be supposed to precede the syncope, as well as that state of relaxation itself, will be followed by amendment: but there is no information, which can lead us to believe, that syncope has often followed the abstraction of *small* quantities of blood, or that success had followed small bleedings. The usual expression is, that after a scanty discharge of blood, it ceased to flow, and that depression of pulse, with *faintness*, not *syncope*, came on, or was increased. We are not informed, whether bleeding has been generally performed in the recumbent, or half erect posture. It is evident from the reports, that the mere act of elevating the body has been followed by faintness and even death: and if venesection has been often attempted in the half erect posture, we may thence account, in some measure, for the frequent want of success in obtaining blood.

"Few remedies, on a fair trial, have been more generally and unequivocally advocated than free bloodletting: and the most that has been urged against it is, that it is not always successful. The advocates for bleeding proceed, however, on the principle, that a certain quantity of blood is to be obtained, in order to ensure success, which few of them estimate at less than about 30 ounces. Those, who are disposed, either less favorably towards bleeding, or to condemn it altogether, object, that if the circulation is in a condition to admit of free bleeding, the case is a mild or favorable one, and would probably yield to other remedies. There is no doubt, that fatal collapse has sometimes suddenly followed, even *large* bleedings, which has staggered the faith of many practitioners in the general safety of the remedy; but, in the great majority of cases, it is after *small* bleedings that this has happened. There is the most ample evidence also, that cases, especially in Europeans, even under the most favorable appearances, will often, in spite of all internal and external remedies, go on to a fatal issue, when bleeding is not practised.

"The cause of collapse coming on after, or during bleeding, in cases of cholera, may perhaps be explained, so as to obviate the objections thence arising against it as a remedy in that disease. It seems unquestionable, that the evacuation of a small portion of blood, such for instance, as we might suppose to be yielded by the remoter branches of vessels, is followed by increased debility: but if we succeed in carrying on the evacuation, till its effects reach the internal vessels and the heart itself, *then* the circulating system seems to be freed from an oppression which impeded its functions, and it becomes equal to the task of propelling the mass of blood. This is a species of indirect excitement. The powers are not raised, but the resistance, or weight is lessened. Such is the theory, which has been adduced in support of bloodletting in cholera. The reader will be aware, that it is not new; its application to the particular disease, of which we are now treating, being the only novelty. *If the theory be true, the presence, or the supervention of collapse, so far from deterring us from going on, should only be regarded as additional reasons for renewing our efforts to get blood.*" lix.

In proof of this, a great many testimonies are brought forward, drawn from the Journals of medical officers, after which Mr. Scot goes on thus :—

"Besides the very ample evidence, which will be found in the printed reports, in favour of bloodletting as a remedy in cholera, the opinions of the majority of Medical Officers whose observations are not inserted, are also decidedly in its favour. The objection chiefly urged, is not against the practice, but rests on the too frequent impracticability of procuring a sufficient quantity of blood. It is acknowledged, even by the most zealous of its advocates, that this difficulty has often occurred, and proved insuperable. When, however, the operation is performed with the moral conviction, that, if successful in obtaining blood, the life of the patient will most probably be saved, the operator will persevere, undiscouraged, in his efforts: he will call in every suitable aid, such as frictions, bathing the arm in hot water, re-opening the orifices of the vessels, administering stimulants, and external warmth. He is not deterred and induced to desist by any intermediate accession of debility, or collapse; nor, is he tempted to rest satisfied with any temporary melioration of pulse: his object goes beyond the present moment, and he feels satisfied, that if he can fully unload the internal vessels, he will save his patient, and if he fails, that he will most probably lose him. It is not considered to be of much importance whether the patient may have been bled before or not, if his present symptoms indicate a repetition of the operation. The principle is, that collapse, in cholera, is not the consequence of the loss of blood, but is a condition which nothing but its abstraction can be trusted to for relieving. In the second case, however, when the medical man is not decided in his own mind, obstacles will be allowed to subdue his fortitude, and arguments will be thence deduced to dissuade him from perseverance. A sudden aggravation of the symptoms of collapse will be regarded as an effect of a remedy, at least doubtful; or, a transient return of vascular action will be held, either as a sufficient advantage gained, or as an indication that bleeding is no longer required. It is thus we may account for a vast number of ineffectual, because undecided attempts at bloodletting; and for a vast number of unsuccessful results, when the quantity abstracted falls short of that, which is required: for the volume of the blood must be reduced in a given proportion, in order to secure the effects expected from it. Any thing short of this will only take away from the patient's strength, and not augment the motive powers of the circulating system; or what is the same thing, will not diminish the resistance to the motive powers.

"Cholera is unquestionably a very dangerous disease, and so many circumstances concur in aggravation of its natural fatality, that we can scarcely hope that any mode of treatment will ever be devised which shall strip it of its formidable character. Much injury has arisen, however, from remedies being brought forward, and tried, as if they were absolutely specific and infallible. And amongst others, bloodletting has been put to this most unjust, and unphilosophical test. If strictly considered, it would, perhaps, be the least of all entitled to the appellation of a specific remedy; for in truth, there is great reason to doubt, whether it be directly curative of the essential symptoms of cholera. Its warmest advocates only consider it as an auxiliary, and never trust to it, but in combination with other, and which indeed appear to be, the strictly appropriate remedies. Congestion, for which alone it is indicated, appears merely to be a symptom or consequence of that morbid state, which forms the first and highest link in the train of cholera actions. The removal of congestion, which is mechanical, allows the heart to respond to the action of the other remedies. Should it be objected, from a consideration of some dissections, that no particular congestion had taken place, the blood appearing to be equally diffused over the vascular system; and that in some instances, the circulation, though ultimately affected, seems to retain its powers for a time, without material depression, yet, even in such cases, venesection, by lessening the volume of the blood, may still have beneficial effects. We should certainly apprehend no danger from it in cases of this description, used, *as it ought invariably to be, in conjunction with the other remedies*." lxi.

These other remedies were principally antispasmodics and stimulants. Of the various means, internal and external, not yet enumerated, it is quite unnecessary to speak. Hot applications to the skin had far less effect than might have been expected from the deadly coldness of the surface, and torpid state of the extreme vessels.

The great body of the work is occupied with a digested history of the progress of the disease, in different places and in different corps—with numerous reports from individual medical officers—and with tabular returns, &c. all of which will be read by the profession in India, with great interest; but would be out of place here. The work, upon the whole, is creditable to the judgment and industry of the author, and entitles him to the thanks of his brethren, Asiatic and British.

